# Supplementary material for: A 3D-Printed Polymer–Lipid-Hybrid Tablet towards the Development of Bespoke SMEDDS Formulations
Source: Pharmaceutics. 2021 Dec 7;13(12):2107. doi: 10.3390/pharmaceutics13122107 (PMC8707116; doi:10.3390/pharmaceutics13122107)
Supplement: Supplementary file 1 [file pharmaceutics-13-02107-s001.zip › pharmaceutics-1466355-supplementary.pdf]

pharmaceutics

Supplementary Materials: A 3D-Printed Polymer–Lipid-Hybrid Tablet Towards the Development of Bespoke SMEDDS Formulations

Bryce W. Barber [1], Camille Dumont [2], Philippe Caisse [2], George P. Simon [3] and Ben J. Boyd [1,4,*]

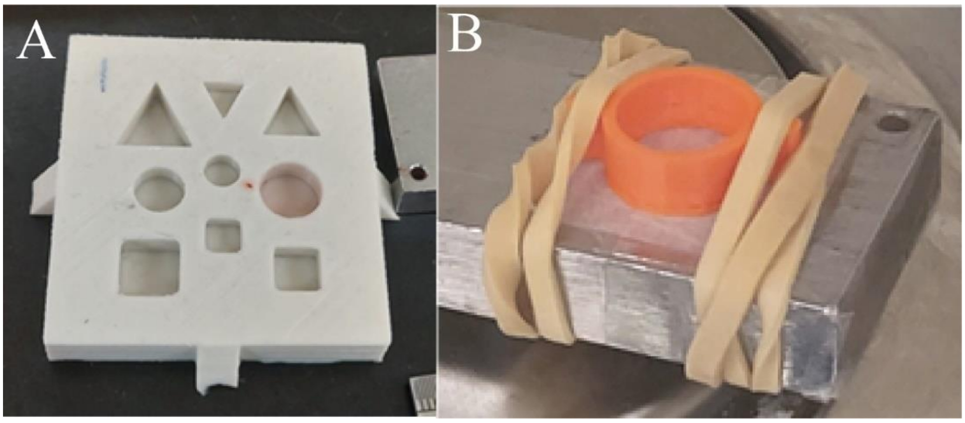

Figure S1. (A) Silicon mould used in generating 'no-scaffold' type systems **(B)** PLA scaffold attached to a steel plate via rubber band used for filling 'dual-face' type systems.

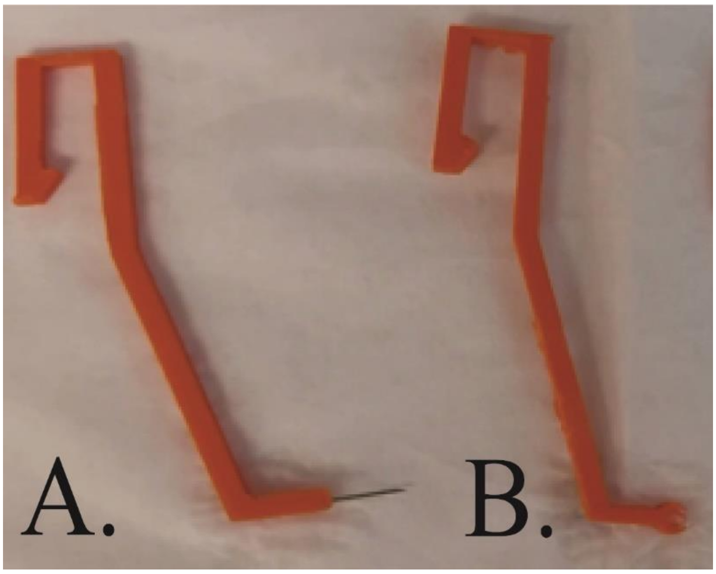

Figure S2. PLA mount variations used for the suspension of tablets in dissolution studies: **(A)** Needle tipped mount; for suspending 'no-scaffold' type tablets **(B)** C-hook tipped mount; for all scaffolded tablets.

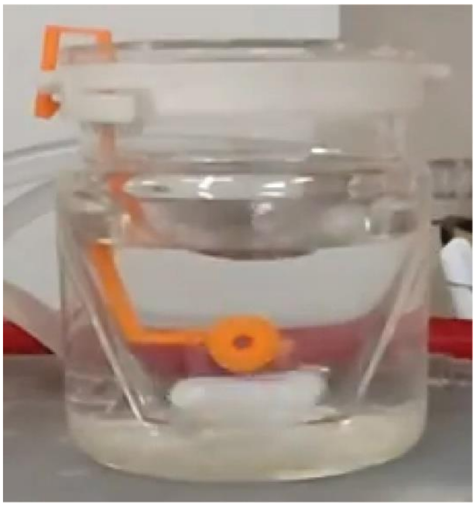

Figure S3. Photograph of a 'semi-open' type scaffolded tablet submerged during dissolution.

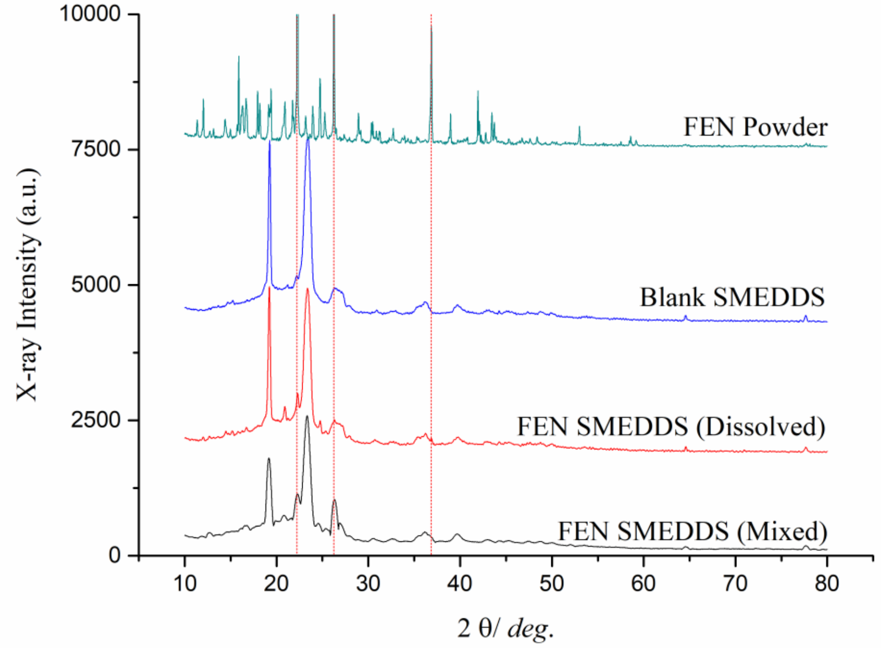

Figure S4. Effect of loading SMEDDS with FEN on the X-ray diffraction profiles of SMEDDS. 'Dissolved' indicates the formulation where the drug was pre-dissolved in the molten SMEDDS, whereas 'Mixed' indicates a physical mixture of powdered solid SMEDDS formulation with the equivalent proportion of crystalline FEN.

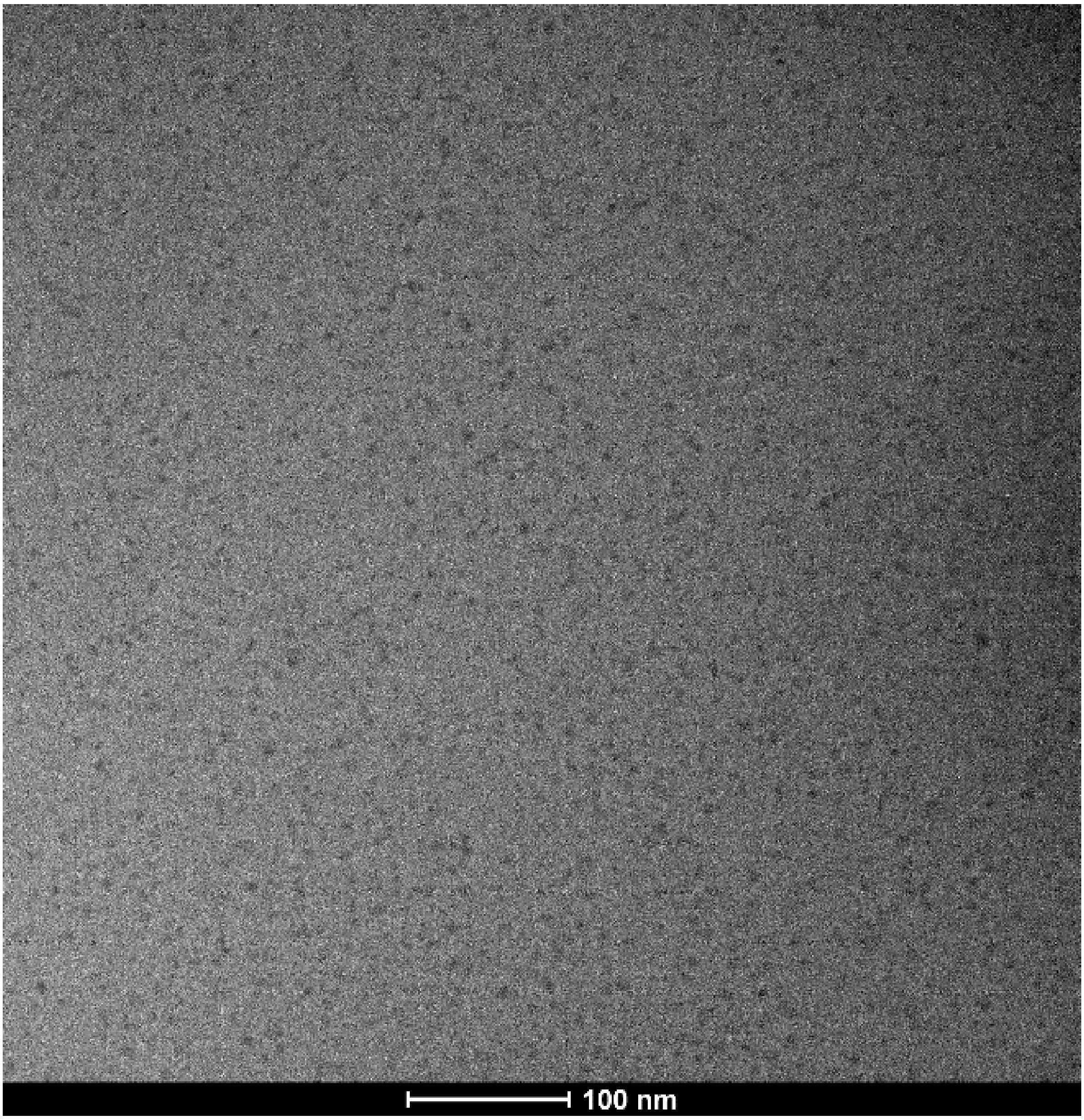

Figure S5. Cryo-TEM image of blank SMEDDS dispersed in water.

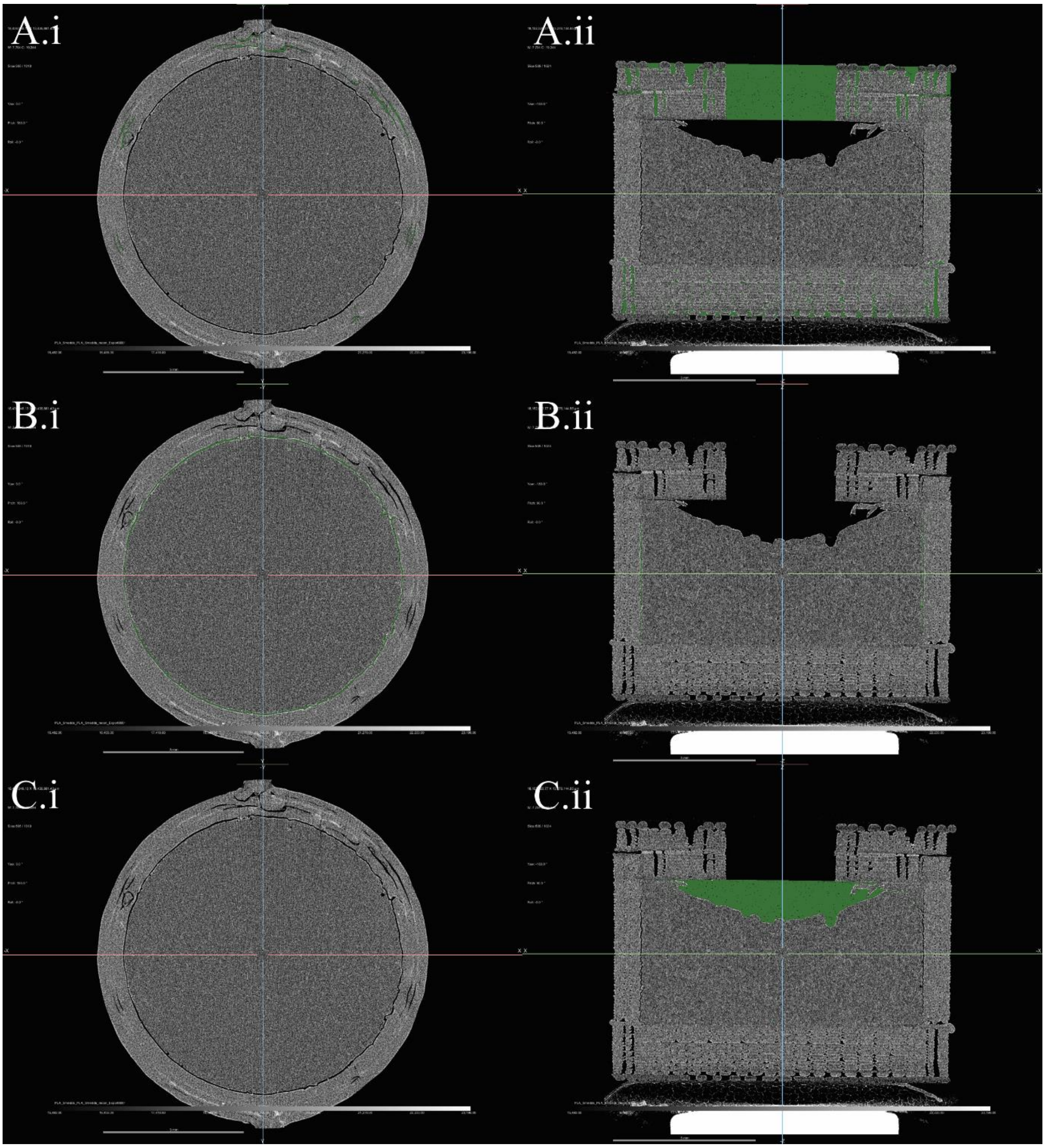

Figure S6. X-Ray CT images of a 'semi-open' type tablet, with the void area highlighted for; **(A)** PLA Scaffold **(B)** Interfacial gap between PLA Scaffold and Lipid formulation **(C)** Lipid formulation. Vertical **(i)** and horizontal **(ii)** slices from the centre of the tablet are displayed.

Table S1. Masses of drug and polymer for scaffold formulations.

Scaffold Composition	Scaffold Type	Formulation Mass (AVG ± %RSD, mg)	Scaffold Mass (AVG ± %RSD, mg)	Total Mass (AVG ± %RSD, mg)	Scaffold % Total Mass
PVOH	No scaffold (NS, *n* = 3)	399.2 ± 0.3%	-	399.2 ± 0.3%	-
	Single face (1F, *n* = 4)	401.0 ± 0.2%	151.8 ± 0.5%	552.9 ± 0.2%	27.4 %
	Double faced (2F, *n* = 4)	401.5 ± 0.3%	221.2 ± 1.5%	622.8 ± 0.7%	35.5 %
	Semi-open (SO, *n* = 4)	401.2 ± 0.2%	319.3 ± 2.8%	717.1 ± 1.1%	44.5 %
	Closed (CL, *n* = 3)	399.8 ± 0.2%	321.6 ± 0.9%	720.3 ± 0.2%	44.6 %
PLA	Single face (1F, *n* = 4)	399.4 ± 0.3%	243.7 ± 4.6%	643.1 ± 1.8%	37.8%
	Double faced (2F, *n* = 3)	400.8 ± 0.4%	502.3 ± 0.1%	903.1 ± 0.9%	55.6%
	Semi-open (SO, *n* = 4)	399.0 ± 0.3%	768.1 ± 4.3%	1167.1 ± 2.9%	65.8%
	Closed (CL, *n* = 3)	398.6 ± 0.1%	801.9 ± 2.5%	1200 ± 1.7%	66.7%

Table S2. Masses of drug and lipid in each compartment of multicompartment systems.

	Multicompartment PLH						
Compartment	A	B	C	D	E	F	Total
Drug (X%)	Clofazimine (7%)	Lumefantrine (7%)	Halofantrine (3.5%)	Clofazimine (7%)	Lumefantrine (7%)	Halofantrine (3.5%)	-
Base formulation	SMEDDS	SMEDDS	SMEDDS	Gelucire 48/16	Gelucire 48/16	Gelucire 48/16	-
Mass.Average ± %RSD (*n* = 4, mg)	60.9 ± 1.4%	62.3 ± 2.1%	63.1 ± 0.7%	61.2 ± 0.8%	64.3 ± 2.4%	61.7 ± 1.2%	373.7 ± 0.7%

Supplementary Material S1: Methods Used in the Quantification of Drug in Dispersion Media

Gradient and isocratic methods were used to separate the model drugs; fenofibrate, halofantrine, lumefantrine and clofazimine.

UPLC analysis was conducted on a Shimadzu CBM-20A HPLC system coupled to; a Shimadzu DGU-20A 5R degasser, a Shimadzu DGU-20A 3R degasser, two Shimadzu LC-30AD solvent delivery pumps, a Shimadzu SIL-30AC MP autosampler, a Shimadzu RF-20A XS fluorescence detector, a Shimadzu SPD-M30A diode array (PDA) detector, analysing at 190 to 800nm.

Method 1: Fenofibrate

A Waters Symmetry C18 column (3.5μm, 100 A, 75 × 4.6mm) was used for analysis in a Shimadzu CTO-20AC column oven, maintained at 40 °C. Separations were conducted using an isocratic method with a flow rate of 1.0ml/min and an injection volume of 50 μL. Employing mobile phases; 0.1 % (*v/v*) formic acid in deionised water (Mobile phase A) and acetonitrile (Mobile Phase B), for a total run time of **10** min. Samples are maintained at 23 °C within the autosampler. A calibration curve was constructed from the following concentrations of fenofibrate; 1, 2, 5, 10, 20, 40 and 80 μg/mL.

Method 2: Clofazimine

A Phenomenex Luna C8(2) column (5μm, 100 A, 150 × 4.6mm) was used for analysis in a Shimadzu CTO-20AC column oven, maintained at 35 °C. Separations were conducted using an isocratic method with a flow rate of 1.0ml/min and an injection volume of 10 μL. Employing mobile phases; 0.25 M sodium acetate trihydrate pH 3.3 (Mobile phase A) and methanol (Mobile Phase B) at a ratio of 26 (%A) : 74 (%B) for a total run time of 6 min. Samples were maintained at 23 °C within the autosampler. A retention time of 3.10 min and detector wavelength of 490 nm was used to integrate clofazimine. A calibration curve was constructed from the following concentrations of clofazimine; 0.3125, 0.625, 1.25, 2.5, 10, 20 and 40 μg/mL.

Method 3: Halofantrine and lumefantrine

A Waters Symmetry C18 column (3.5μm, 100 A, 75 × 4.6mm) was used for analysis in a Shimadzu CTO-20AC column oven, maintained at 35 °C. Separations were conducted using a gradient method with a flow rate of 1.0 mL/min and an injection volume of 20 μL. Employing mobile phases; 30 mM sodium hexane sulfonate (Mobile phase A) and methanol (Mobile Phase B) for a total run time of 15 min. Samples were maintained at 23 °C within the autosampler. A retention time of; 7.29 min and 8.52 min; and detector wavelength of; 257 nm and 236 nm, was used to integrate halofantrine and lumefantrine respectively. A calibration curve was constructed from the following concentrations of halofantrine and lumefantrine in parallel; 0.3125, 0.625, 1.25, 2.5, 10, 20 and 40 μg/mL.
